# Inter-individual Variability in Responses to 7 Weeks of Plyometric Jump Training in Male Youth Soccer Players

**DOI:** 10.3389/fphys.2018.01156

**Published:** 2018-08-20

**Authors:** Rodrigo Ramirez-Campillo, Cristian Alvarez, Paulo Gentil, Jason Moran, Felipe García-Pinillos, Alicia M. Alonso-Martínez, Mikel Izquierdo

**Affiliations:** ^1^Department of Physical Activity Sciences, Research Nucleus in Health, Physical Activity and Sport, Laboratory of Measurement and Assessment in Sport, Universidad de Los Lagos, Osorno, Chile; ^2^Faculdade de Educacao Fısica e Danca, Federal University of Goiás, Goiania, Brazil; ^3^Department of Sport, University Centre Hartpury, University of the West of England, Bristol, United Kingdom; ^4^Department of Physical Education, Sports and Recreation, Universidad de La Frontera, Temuco, Chile; ^5^Department of Health Sciences, Navarrabiomed, CIBER of Frailty and Healthy Aging (CIBERFES), Instituto de Salud Carlos III, Pamplona, Public University of Navarra, Navarra, Spain

**Keywords:** football, force-velocity curve, jump training, stretch-shortening cycle, maturation, strength

## Abstract

The purpose of this study was to compare the inter-individual variability in the effects of plyometric jump training (PJT) on measures of physical fitness (sprint time, change of direction speed, countermovement jump, 20- and 40-cm drop jump reactive strength index, multiple five bounds distance, maximal kicking distance, and 2.4-km time trial) in youth soccer players who completed a PJT program versus players who completed soccer training only. In a single-blinded study, participants aged between 10 and 16 years were randomly divided into a PJT group (*n* = 38) and a control group (*n* = 38). The experimental group participated in a PJT program twice weekly for 7 weeks, whereas the control group continued with their regular soccer training sessions. Between-group differences were examined using a Mann–Whitney U test. Nonresponders where defined as individuals who failed to demonstrate any beneficial change that was greater than two times the typical error of measurement from zero. The results indicated that the mean group improvement for all physical fitness measures was greater (*p* < 0.05) in the PJT group (Δ = 0.4 to 23.3%; ES = 0.04 to 0.58) than in the control group (Δ = 0.1 to 3.8%; ES = 0.02 to 0.35). In addition, a significantly greater (*p* < 0.05) number of responders across all dependent variables was observed in the PJT group (from 4 up to 33 responders) than in the control group (from 0 up to 9 responders). In conclusion, compared to soccer training only, PJT induced greater physical fitness improvements in youth soccer players, with a greater number of responders for all the physical fitness tests related to jumping, speed, change of direction speed, endurance, and kicking technical ability.

## Introduction

The habitual development of “athleticism” to improve health, enhance physical fitness, reduce the relative risk of injury, and develop the confidence and competence of youths is particularly relevant in programs for children and adolescents (Lloyd et al., 2014) into the holistic long-term athletic development process in youths. Of particular relevance among training programes for youth soccer players is resistance training, a specialized method of conditioning that involves the progressive use of a wide range of resistive loads, including body mass, and a variety of training modalities (e.g., machine-based training, free weight training, plyometric training, complex training, and functional training) to enhance muscular fitness and athletic performance (Behm et al., 2008; Granacher et al., 2016; Barbalho et al., 2018). Plyometric jump training (PJT) is a common resistance training modality that incorporates the stretch-shortening cycle of muscles to acutely improve the rate of force development, with the long-term aim to induce neuromuscular adaptations (Markovic and Mikulic, 2010; Lloyd et al., 2014). The PJT programs have the added advantages of not requiring expensive equipment or large spaces and have been shown to be an enjoyable (Ward et al., 2007) and effective form of training for youth soccer players (Bedoya et al., 2015), inducing physical fitness improvements such as jumping, sprinting, kicking, and change of direction, key traits for soccer (Barnes et al., 2014). These actions might precede most of the goals scored in competitive leagues (Faude et al., 2012) and may correlate with competition success (Arnason et al., 2004). Repeating these maximal-intensity actions across a game is also important (Carling et al., 2012) and might be associated with endurance (Helgerud et al., 2001), which also may be enhanced with PJT in youth soccer players (Ramirez-Campillo et al., 2014b, 2015a,b). On this basis, PJT programs have received extensive attention from researchers in recent years (Ramirez-Campillo et al., 2018a).

However, despite extensive investigation, research articles usually report the group response (i.e., the mean change within a training group) from youth soccer players to PJT without considering the wider inter-individual variability in the response to exercise training (IVRET), in which participants can be broadly classified into two types: responders and nonresponders (NR) (Alvarez et al., 2017a,b,c, 2018a). Additionally, among the few studies of IVRET, most have focused on cardiorespiratory fitness and metabolic measures without considering other measures more relevant to soccer players (i.e., jumping) (Arnason et al., 2004), which not only may exhibit an IVRET but also may show a different response to training in each individual over time (Piirainen et al., 2014). Thus, it is important to not only study the IVRET phenomenon in a single variable but also study a cluster of dependent variables relevant to each study population (Barbalho et al., 2017).

In addition to the above, most of the cited studies applied endurance training stimulus only or a combination of endurance and resistance training. Moreover, most investigations were carried out in adult populations. As the effects of resistance training and PJT may differ between individuals according to their development (Asadi et al., 2017; Moran et al., 2017a,b,c), the IVRET phenomenon typically observed in adults may be different from that in youth populations. Therefore, individual responsiveness to PJT alone remains a phenomenon that warrants further exploration. To our knowledge, only one study has analyzed the IVRET phenomenon after plyometric training (Radnor et al., 2017). However, the study was not on youth soccer players, and only sprint and jumping variables were analyzed.

The purpose of this study was to compare the IVRET of physical fitness measures (jumping, reactive strength index, speed, change of direction ability, kicking performance, and endurance) in youth soccer players who completed a PJT program or soccer training only. According to relevant literature (Radnor et al., 2017), it was hypothesized that a higher number of responders – based on measures of physical fitness – would be observed among youth soccer players after PJT than among those who underwent soccer training only.

## Materials and Methods

### Participants

**FIGURE 1:**
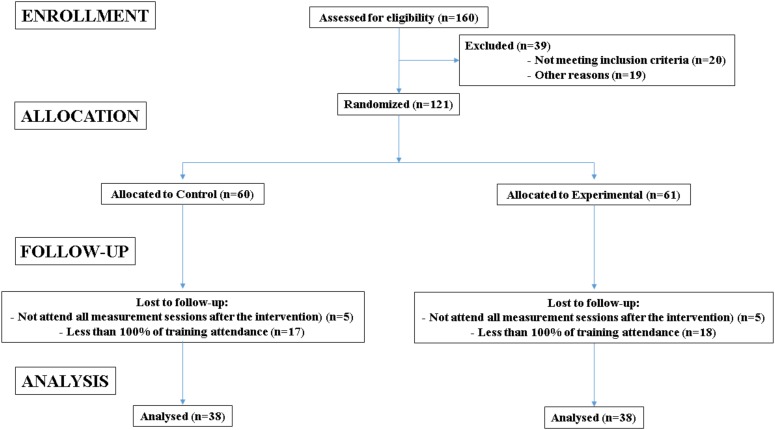
CONSORT diagram of the full recruitment and randomization process.

Seventy-six male soccer players aged between 10 and 16 years (control group: Tanner stage 3.7 ± 1.1; body mass index, 19.9 ± 2.3 kg⋅m^-2^; PJT group: Tanner stage, 3.7 ± 1.1; body mass index, 19.9 ± 1.7 kg⋅m^-2^) volunteered to participate in the study. All participants had previously been engaged in soccer, with (i) more than 2 years of systematic soccer training and competition experience and (ii) continuous soccer training in the last 6 months. Although the participants regularly performed sporadic jumps during training and competition, they had not systematically performed PJT in the 6 months prior to this study and had no history of regular strength training. **Figure 1** depicts the CONSORT diagram of the full recruitment and randomization process. Participants were divided into a PJT group (*n* = 38) or a control group (*n* = 38). The experimental group participated in a PJT program twice weekly for 7 weeks, whereas the control group carried out their regular soccer training sessions only. Participants were reminded during each training session to maintain their usual physical activity habits during the experiment. Exclusion criteria included subjects with (a) potential medical problems or a history of ankle, knee, or back pathology in the 3 months before the study, (b) medical or orthopedic problems that compromised their participation or performance in the study, (c) any lower extremity reconstructive surgery in the past 2 years or unresolved musculoskeletal disorders, and (d) subjects who were taking or had previously taken anabolic steroids, growth hormone, or related performance-enhancement drugs of any kind. However, individuals were not excluded if they had been taking vitamins, minerals, or related natural supplements (other than creatine monohydrate).

The following dependent variables were tested in all participants before and after the 7-week intervention: 20-m sprint time, change of direction speed test time (CODS), countermovement jump (CMJ) height, 20- (RSI20) and 40-cm (RSI40) drop jump reactive strength index, multiple 5 bounds distance (MB5), maximal kicking test for distance (MKD), and 2.4-km time trial. Following previous criteria (Alvarez et al., 2018b), NR to each of the dependent variables were defined as individuals who failed to demonstrate an increase or decrease (in favor of beneficial changes) that was greater than two times the typical error of measurement (TE) away from zero. Parental informed consent and participant assent were obtained in advance of the study. The Department Research Ethics Committee, in accordance with the Declaration of Helsinki, approved the study.

Sample size was computed according to the changes observed in plyometric (i.e., reactive strength index) performance (*d* = 0.3 mm⋅ms^-1^; *SD* = 0.35) in a group of young adolescents submitted to the same training program (Ramirez-Campillo et al., 2013, 2014b). Eight participants per group would yield a power of 80% and α = 0.05.

### Experimental Design

Subjects followed a 90-min familiarization session before testing to reduce any learning effects, and a warm-up was completed at the beginning of each testing session (Andrade et al., 2015). Standardized tests were scheduled >48 h after competition or high-intensity physical training to minimize the influence of fatigue. All tests were performed over 2 days under similar weather, time, and field conditions before and immediately after the 7-week period. On day one, the players’ physical characteristics (height, body mass, and self-assessed pubic hair and genital stage) were assessed, and physical fitness tests were conducted in the following order: CMJ, RSI20 and RSI40, MB5, 20-m, and CODS test. On day two, the MKD and a 2.4-km time trial were performed. All tests were administered in the same order before and after training, with players wearing the same sporting attire. Data were recorded by the same investigators who were blinded to the group allocation of the participants. In addition, all participants (and their parents or guardians) were instructed to have a good night’s sleep (≥9 h) before each testing day and to be well hydrated. A standardized meal rich in carbohydrates was provided to the participants 2–3 h before measurements. All participants were motivated to give their maximum effort during physical fitness measurements. At least 2 min of rest were allowed between each trial to reduce effects from fatigue. While waiting, the participants performed low-intensity activity to maintain physiological readiness for the next test. The best score of three trials was recorded for all physical fitness tests, apart from the single 2.4-km time trial, which was performed just once. As in previous studies that used similar procedures (Ramirez-Campillo et al., 2013, 2014b), high intra-class correlation coefficients were obtained for the different physical fitness tests, varying between 0.81 and 0.98.

### Somatic and Maturity Measures

Height was measured using a wall-mounted stadiometer (Butterfly, Shanghai, China) recorded to the nearest 0.5 cm. Body mass was measured to the nearest 0.1 kg using a digital scale (BC-554 Ironman Body Composition Monitor; Tanita, IL, United States). Body mass index was then calculated (kg⋅m^-2^). Maturity was determined by self-assessment of Tanner stage as previously outlined (Ramirez-Campillo et al., 2014b).

### Vertical Jump Tests

Testing included the execution of maximal CMJ, RSI20, and RSI40. All jumps were performed on a mobile contact mat (Ergojump; Globus, Codogne, Italy) with arms akimbo. Take-off and landing was standardized to full knee and ankle extension on the same ground position. The participants were instructed to maximize jump height and minimize ground contact time during the RSI20 and RSI40 after descending from 20- and 40-cm boxes, respectively. The RSI was calculated as previously reported (Ramirez-Campillo et al., 2014b), dividing jumping height (mm) by time contact (ms), thus expressed in mm⋅ms^-1^.

### Multiple Five Bounds Test

The MB5 was started from a standing position from which participants performed a set of five forward jumps with alternative left- and right-leg contacts to cover the longest distance possible. The distance of the MB5 was measured to the nearest 0.5 cm using a tape measure (Ramirez-Campillo et al., 2014b). Participants were motivated to give their maximum effort during three trials, with ∼2 min of rest between trials. Considering its specificity in soccer players, the test is an adequate alternative to vertical jumps as a measure of explosive strength and coordination (Diallo et al., 2001; Meylan and Malatesta, 2009; Ramirez-Campillo et al., 2014b).

### Twenty-Meter Sprint and Change of Direction Speed Test

The sprint time was measured to the nearest 0.01 s using single beam infrared reds photoelectric cells (Globus Italia, Codogne, Italy). The starting position was standardized to a still split standing position with the toe of the preferred foot forward and behind the starting line. The sprint start was given by a random sound, which triggered timing. The photoelectric signal was positioned at 20 m and set ∼0.7 m above the floor (i.e., hip level) to capture the trunk movement rather than a false trigger from a limb. The CODS test has been described previously, and its reliability addressed elsewhere (Ramirez-Campillo et al., 2014b). The timing system and procedures were the same as the 20-m sprint with the exception that subjects started lying on their stomach on the floor with their face down.

### Maximal Kicking Distance Test

After a standard warm-up, each player kicked a new size 5 soccer ball (Nike Seitiro, FIFA certified) for maximal distance on a soccer field. Two markers were placed on the ground side by side to define the kick line. Participants performed a maximal instep kick with their dominant leg after a run up of two strides. A 75-m metric tape was placed between the kicking line and across the soccer field. An assessor was placed near the region where the ball landed after the kick to mark the point of contact and to measure the distance kicked. The distance was measured to the nearest 0.2 m. All measurements were completed with a wind velocity <20 km⋅h^-1^ (local Meteorological Service). Previous studies have reported a high level of reliability for similar soccer kicking tests (Ramirez-Campillo et al., 2014b).

### Time Trial 2.4-km Test

As previously recommended (Ramirez-Campillo et al., 2014b; Assuncao et al., 2017), the time-trial 2.4 km test was used considering its multiple facet requirement (maximal oxygen consumption, lactate threshold, running economy, muscle power) (Coyle, 1995), likely to affect aerobic-related performance in soccer. After a warm-up run of 800-m and 4 min of rest, players performed six laps of a 400-m outdoor dirt track, timed to the nearest second, with a stopwatch. The wind velocity at all times was ≤8.9 km⋅h^-1^, the relative humidity was between 50 and 70%, and the temperature was between 15 and 20°C (local Meteorological Service). Motivation was considered maximal as the test was conducted as part of the team selection process.

### Training Intervention

This study was completed during the mid-portion of the players’ competition period. Before this period, participants completed 2 months of summer pre-season training, were three 90 min training sessions were completed per week. The control group did not perform PJT but did perform their usual soccer training, which included 20 min of technical drills, 20 min of tactical drills, 20 min of small-sided games, and 30 min of simulated competitive games per session. In addition, once a week, injury prevention drills were incorporated. To ensure that training loads were similar between groups, the session rating of perceived exertion (RPE) was determined by multiplying the soccer training duration (in minutes) by session RPE, as previously described in studies of young soccer players (Ramirez-Campillo et al., 2014b). Before the initiation of the training period, participants from the PJT group were instructed on proper execution of all the exercises included in the program. During the intervention, the PJT group replaced some technical drills (e.g., ball heading exercises) with plyometric drills within the usual 90-min practice period, twice per week for 7 weeks. This training program has been shown to induce significant physical fitness adaptations in youth soccer players during the in-season period as part of a replacement for some low-intensity technical drills (Ramirez-Campillo et al., 2014b). All plyometric sessions lasted ∼21 min and were performed just after the warm-up to ensure that the players were in a rested state and that they gained optimal benefits from the specific program (Ramirez-Campillo et al., 2018).

Briefly, the PJT included 60 drop jump repetitions per session and was performed on a grass soccer field. The athletes completed three sets of 10 repetitions from 20-, 40-, and 60-cm height boxes, in a random schedule, in order to maximize adaptations (Hernández et al., 2018), for a total of 840 foot contacts after 7 weeks of training. The participants were instructed to jump as high and fast as they could, with maximal voluntary effort (Ramirez-Campillo et al., 2018b), for each repetition. We did not increase the training volume during the 7-week period, as we used high-intensity plyometric exercises performed with maximal effort; however, an adequate training stimulus was applied during each plyometric session, as previously demonstrated in youth soccer players (Ramirez-Campillo et al., 2014a,b). The rest period between repetitions and sets was ∼15 and ∼90 s (Ramirez-Campillo et al., 2013, 2014b), respectively. Previous research has demonstrated that this is an adequate rest interval for this type of training (Ramirez-Campillo et al., 2014b). As players did not have any history of formal PJT, all exercises were supervised with an investigator-to-participant ratio of 1:6. A high investigator-to-participant ratio have demonstrated greater benefits during explosive resistance training interventions (Ramirez-Campillo et al., 2017). Particular attention was paid to exercise demonstration and execution, providing maximal motivation to athletes during each jump. Training sessions were separated by a minimum period of 48 hours (including games). Aside from the formal training intervention, all participants attended their regular physical education classes.

The reliability of jump heights and contact times for the PJT drills was verified in a randomly assigned subsample of participants (i.e., *n* = 2) during two randomly selected training sessions. During these sessions, ground contact-times and jump heights were tested using the same procedures and equipment as described above. Briefly, the maximal intensity for drop jumps was verified by measuring height and contact-time of the respective drill.

### Statistical Analysis

The between group differences in percentage change for all physical fitness variables was examined using a Mann–Whitney U test. Percentage change from baseline testing was calculated for all individuals in each of the physical fitness variables. The NR were identified and defined as individuals who failed to demonstrate an increase or decrease (in favor of beneficial changes) in physical fitness that was greater than two-times the TE away from zero, calculated using a previously stablished equation (Bonafiglia et al., 2016). For the current study, three repeats of each physical fitness test were used in order to calculate the TE. A change beyond two times the TE was representative of a high probability (i.e. 12 to 1 odds) that the observed response was a true physiological adaptation beyond what might be expected to result from technical and/or biological variability. Thus, the TE were the following [CMJ, 0.074 (cm) × 2; RSI20, 0.00054 (mm/ms) × 2; RSI40, 0.00052 (mm/ms) × 2; MB5, 0.017 (m) × 2; 20 m, 0.0074 (s) × 2; CODS, 0.038 (s) × 2; MKD, 0.106 (m) × 2]. For the 2.4 km time trial test, considering that only one maximal attempt was employed during testing, the criteria to determine NR were those athletes that did not reduce the total time in the test. Additionally, the Chi-Square test (*X*^2^) was used for comparisons between groups of subjects who were into the 2 × TE calculated in each outcome (NR), or beyond two times the TE [responders (R)]. Cohen’s *d* effect sizes (ES) were calculated for within groups changes in physical fitness and interpreted using previously outlined ranges (<0.2 = trivial; 0.2–0.6 = small; 0.6–1.2 = moderate; 1.2–2.0 = large; 2.0–4.0 = very large; >4.0 = extremely large).

## Results

No differences were observed between the PJT and the control groups in the somatic and maturity measures, nor before nor after the intervention.

At baseline, no differences were observed between the groups for all the dependent variables, with values for the whole group of players being 26.8 ± 5.2 cm for CMJ, 0.102 ± 0.04 mm⋅ms^-1^ for RSI20, 0.103 ± 0.04 mm⋅ms^-1^ for RSI40, 8.9 ± 1.2 m for MB5, 4.35 ± 0.5 s for 20 m, 20.2 ± 2.8 s for CODS, 10.6 ± 0.8 min for 2.4-km time trial, and 31.8 ± 7.6 m for the MKD test.

**FIGURE 2:**
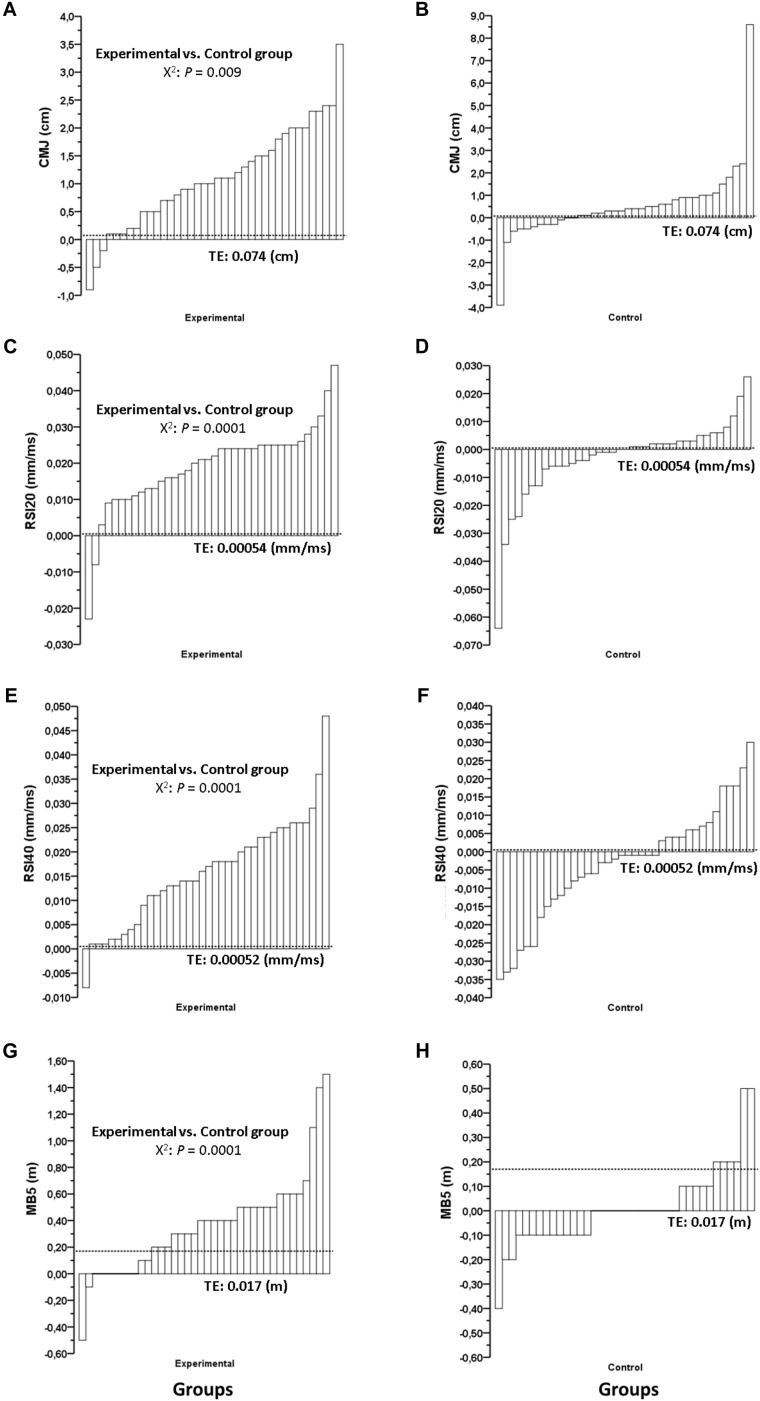
Effects of 7 weeks of plyometric jump training plus soccer (Experimental) and only soccer training (Control) on individual pre-post change for **(A,B)** countermovement jump, **(C,D)** 20-cm reactive strength index, **(E,F)** 40-cm reactive strength index, and **(G,H)** multiple 5 bounds test. Note: all significant *p-*values (<0.05) denote a greater number of responders in the Experimental group compared to the Control group. Responders were identified on an individual basis according to the typical error of measurement (TE), represented by a dotted line. *X*^2^, chi-squared test.

**FIGURE 3:**
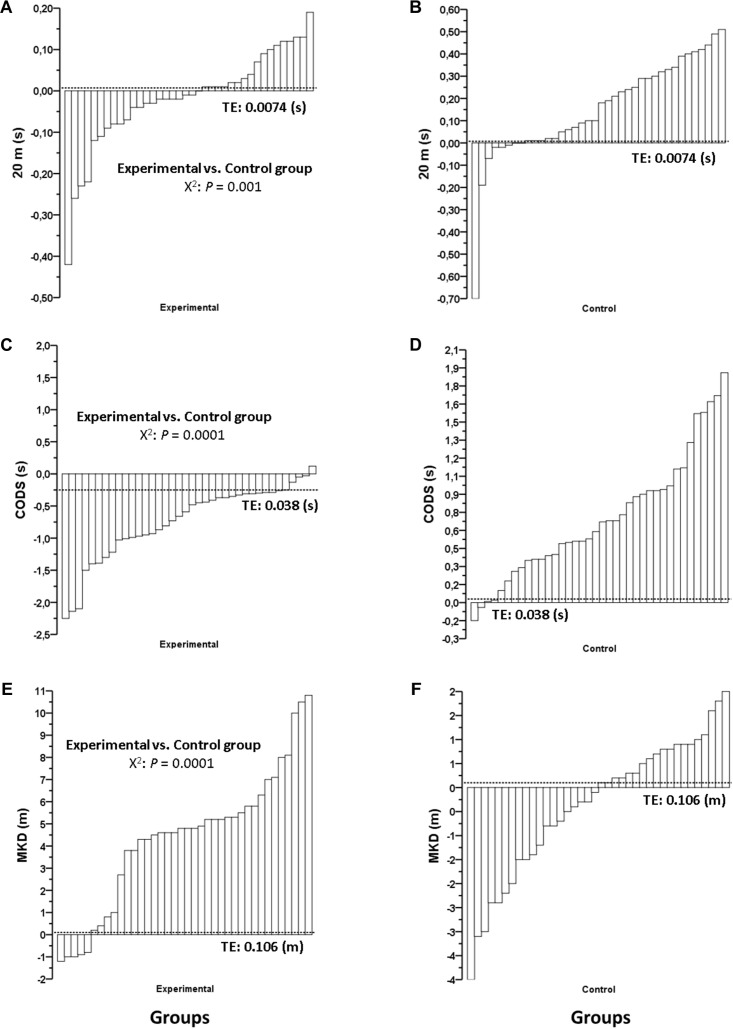
Effects of 7 weeks of plyometric jump training plus soccer (Experimental) and only soccer training (Control) on individual pre-post change for **(A,B)** 20-m sprint time (20 m), **(C,D)** change of direction speed (CODS), and **(E,F)** maximal kicking test for distance (MKD). Note: all significant *p*-values (<0.05) denote a greater number of responders in the Experimental group compared to the Control group. Responders were identified on an individual basis according to the typical error of measurement (TE), represented by a dotted line. *X*^2^, chi-squared test.

**Table 1** shows the mean group response to each intervention and the significant between-group differences in percentage change for all physical fitness variables. For CMJ, RSI20, RSI40, MB5, 20-m, CODS, 2.4-km time trial, and MKD physical fitness variables, a significantly greater (*p* < 0.05) improvement was observed in the PJT group (ES = 0.21, 0.58, 0.37, 0.28, 0.04, 0.27, 0.28, 0.53, respectively) when compared to the control group (ES = 0.13, 0.08, 0.06, 0.01, 0.35, 0.25, 0.04, 0.06, respectively). The individual change in absolute units per each physical fitness test is shown in **Figures 2, 3**.

**Table 1:** Effects of 7 weeks of plyometric jump training plus soccer (Experimental) and only soccer training (Control) on mean group pre-post change (group % change) and number of responders (R) for performance variables.

	Experimental (*n* = 38)		Control (*n* = 38)	
		
	Group % change	*R, n*	Group % change	*R, n*
Countermovement jump (cm)	4.4 ± 3.8^∗^	18^†^	2.4 ± 7.1	9
20-cm reactive strength index (mm⋅ms^-1^)	23.3 ± 17.3^†^	33^†^	-1.7 ± 13.2	5
40-cm reactive strength index (mm⋅ms^-1^)	16.7 ± 13.2^†^	29^†^	-1.0 ± 17.3	7
5 multiple bounds (m)	4.2 ± 4.8^†^	24^†^	0.1 ± 2.0	3
20-m sprint time (s)	-0.4 ± 2.7^†^	4^†^	3.8 ± 5.3	2
Change of direction speed test (s)	-3.5 ± 2.5^†^	19^†^	3.6 ± 2.5	0
2.4-km time trial (min)	-1.9 ± 2.4^†^	19^†^	-0.3 ± 1.9	6
Maximal kicking distance test (m)	14.0 ± 10.7^†^	29^†^	-1.4 ± 5.2	3


*^†^Significantly greater than Control (p < 0.01); ^∗^significantly greater than Control (p < 0.05).*

When responders in the PJT group were compared to those in the control group, the chi-squared analysis revealed a significantly greater number of responders after PJT for CMJ, RSI20, RSI40, MB5, 20-m, CODS, 2.4-km time trial, and MKD (**Table 1**).

Specifically, for the CMJ test, 47% of the players were identified as responders in the PJT group, compared to only 24% in the control group. For the RSI20 test, 87% of the players were identified as responders in the PJT group, compared to only 13% in the control group. For the RSI40 test, 76% of the players were identified as responders in the PJT group, compared to 18% in the control group. In the MB5 test, 63% of the players were identified as responders in the PJT group, compared to 8% in the control group. For the 20-m test, 11% of the players were identified as responders in the PJT group, compared to 5% in the control group. In the CODS test, 50% of the players were identified as responders in the PJT group, while none of the players from the control group demonstrated a response. Regarding the 2.4-km time trial test, 50% of players were identified as responders in the PJT group, compared to only 16% in the control group. In the MKD test, the percentage of responders for the PJT and the control groups were 76% vs. 8%, respectively.

## Discussion

The purpose of this study was to compare the IVRET on measures of physical fitness in youth soccer players who completed PJT versus players who completed soccer training only. The main findings indicate that there was a greater number of responders in the PJT group than in the control group across all variables measured. Thus, the combination of PJT with soccer training induced a greater number of responders than did soccer training only in measures of jumping, speed, change of direction, endurance, and technical abilities in youth soccer players. Current results contribute novel findings regarding the IVRET phenomenon in youth soccer players after a PJT program. The IVRET analysis carried out in the current study may help better assess results from PJT interventions for improved individualization of training approaches.

The current findings show larger improvements in jumping performance in the PJT group than in the control group. These results corroborate previous findings showing that PJT was effective in improving jumping performance in youth soccer players (Bedoya et al., 2015). Improvements in jumping height ability may be a relevant aim for soccer players, since a greater jumping ability may be related to a better position in a competitive league (Arnason et al., 2004), and therefore, the integration of PJT in the regular training schedules of youth players may be an effective method of enhancing competitiveness. The improvement observed in the PJT group may have been induced by increased neural drive to the agonist muscles, improved intermuscular coordination, changes in musculotendinous mechanical stiffness characteristics, changes in muscle size or architecture, and changes in single-fiber mechanics (Markovic and Mikulic, 2010). However, without specific physiological measurements, only speculative conclusions are possible. In a previous study (Radnor et al., 2017), although several resistance training methods were applied in youths, only PJT was associated with a greater number of responders in jumping performance (i.e., RSI) than that in the control group. Our results also showed a greater number of responders in jumping performance in the PJT group (*n* = 18–33, depending on the jump test) than in the control group (*n* = 3–9). In addition, our findings expanded previous knowledge, showing that PJT induced a greater number of responders than soccer training only for variables related to vertical jumping, horizontal jumping, acyclical jumping (i.e., CMJ; RSI), and cyclical jumping (i.e., five multiple bounds). Of note, a greater number of responders was observed for jumping actions that involved fast stretch-shortening cycle (SSC) measures (i.e., RSI) than for those involving slow SSC measures. This result may reflect the specific effect of the training program, as only drop jumps were implemented in the current study, similar to previous studies (Ramirez-Campillo et al., 2015a,b). Given the relevance of the rate of force development for youth soccer long-term athletic development (Meylan et al., 2014), the observed improvement in RSI could enhance physical qualities related to game performance.

Regarding the 20-m sprint test, our results indicate that the change in sprinting time was greater in the PJT group than in the control group after 7 weeks. Previous findings confirm that PJT may increase sprint performance (Saez de Villarreal et al., 2012; Assuncao et al., 2017). Moreover, a larger number of responders was observed in the PJT group (*n* = 4) than in the control group (*n* = 2). However, it must be noted that the improvement in the 20-m test was rather small (-0.4%) compared to the improvements in other physical fitness variables, possibly owing to the lack of motor pattern similarity between the training stimulus (i.e., vertical) and the sprinting performance test. Therefore, PJT may be the best complement to other methods for inducing positive adaptations that improve sprint performance. Previous studies have also called for more specific training methods to improve sprint performance in youth soccer players (Ramirez-Campillo et al., 2014b, 2015b), especially when the PJT stimulus was of a vertical nature, given the importance of horizontal force production and its relevance to sprint performance (Morin et al., 2012).

An improvement in the CODS test was observed in the PJT group compared to the control group. Performance in this test is commonly improved after PJT programs in youths (Asadi et al., 2017), including youth soccer players (Ramirez-Campillo et al., 2014b; Bedoya et al., 2015). Several underlying factors may help explain the improvements in CODS performance, such as improved muscle power and concentric and eccentric muscle strength (Young et al., 2015). To our knowledge, this was the first study to report and compare responders and NR to PJT in CODS performance among youth soccer players. Our results indicate that the PJT program induced 19 responders; meanwhile, no responders were detected in the control group. These results suggest the advantage of including some specific jump drills in the regular training schedule of youth soccer players to help them perform the CODS movements that commonly occur during a competitive soccer match (Stolen et al., 2005). Moreover, CODS is an important determinant of high performance throughout the course of a soccer player’s career and, therefore, must be developed from a young age (le Gall et al., 2010).

Regarding the 2.4-km time trial test, the PJT induced an improvement in this test in the youth soccer players. Improvements in similar tests have been previously reported in youth soccer players after PJT (Ramirez-Campillo et al., 2014b; Assuncao et al., 2017). In addition to the improvements in endurance performance in the PJT group, the number of responders in the PJT group reached 50%, which is considerably higher than the 16% in the control group. The positive effects of PJT on performance in the 2.4-km time trial test might be due to the running economy associated with PT (Barnes and Kilding, 2015), which may have offset fatigue and allowed the athletes to maintain a higher velocity during the test.

In the kicking performance test, the PJT group experienced greater improvement than the control group. This is a particularly interesting observation considering that the PJT group replaced some technical low-intensity soccer drills with high-intensity jumping actions. Moreover, a greater number of responders (*n* = 29) was observed after PJT than after soccer training only (*n* = 3). These results are similar to those previously reported (Michailidis et al., 2013). Considering that players had 2 or more years of soccer experience, improvements in kicking performance in the PJT group were probably not related to changes in technical ability and were more likely due to improvements in neuromuscular (Markovic and Mikulic, 2010) and biomechanical adaptations induced by PJT (Lees et al., 2010).

Although with several strengths, some potential limitations should be acknowledge. First, we did not obtain physiological assessments to better understand the underlying mechanisms of PJT induced adaptations in responders and NR athletes. However, physical fitness tests (i.e., jumping) are significantly and highly associated with physiological [i.e., type of muscle fiber (Bosco and Komi, 1979)] and biomechanical parameters (Ham et al., 2007; Chamari et al., 2008; Meylan et al., 2010) as well as with sporting success. The latter is most important for athletic cohorts (Arnason et al., 2004; Wisloff et al., 2004). Second, although the replacement of technical drills during the in-season period in youth soccer players is uncommon, our approach did not induced a negative impact on the player’s technical abilities. In fact, our results proved that athletes in the PJT group improved their ability to kick a soccer ball. However, from an ecological valid point of view, although PJT can improve physical fitness in youth male soccer players, to optimize training adaptations, this training strategy should be adequately applied in a more complex training plan that incorporates other explosive (e.g., sprints), endurance, technical, and tactical-oriented training methods. Future studies should aim to obtain physiological assessments to better understand the underlying mechanisms of PJT induced adaptations in responders and NR athletes. Moreover, future studies should aim to analyze the IVRET according to the maturity of soccer players, including both female and males athletes.

In conclusion, compared to soccer training only, PJT induced greater physical fitness improvements in youth soccer players, with a greater number of responders in the PJT group in all the physical fitness tests related to jumping, speed, change of direction, endurance, and kicking technical ability. The current results contribute novel findings regarding the IVRET phenomenon in youth soccer players after a PJT program. The IVRET analysis carried out in the current study may help to better assess results from PJT interventions for improved individualization of training approaches.

## Author Contributions

RR-C, CA, and MI designed the work. RR-C and CA acquired the data. PG, JM, AMA-M, and CA analyzed and interpreted the data. RR-C, CA, and FG-P drafted the work. All authors critically revised the work, approved the final version to be published, and agreed to be accountable for all aspects of the work in ensuring that questions related to the accuracy or integrity of any part of the work were appropriately investigated and resolved.

## Conflict of Interest Statement

The authors declare that the research was conducted in the absence of any commercial or financial relationships that could be construed as a potential conflict of interest.
